# Exome Sequencing and Gene Prioritization Correct Misdiagnosis in a Chinese Kindred with Familial Amyloid Polyneuropathy

**DOI:** 10.1038/srep26362

**Published:** 2016-05-23

**Authors:** Hui Chen, Xueya Zhou, Jing Wang, Xi Wang, Liyang Liu, Shinan Wu, Tengyan Li, Si Chen, Jingwen Yang, Pak Chung Sham, Guangming Zhu, Xuegong Zhang, Binbin Wang

**Affiliations:** 1 Department of Neurology, Military General Hospital of Beijing PLA, Beijing, China; 2 MOE Key Laboratory of Bioinformatics, Bioinformatics Division and Center for Synthetic and Systems Biology, TNLIST/Department of Automation, Tsinghua University, Beijing, China; 3 Department of Psychiatry and Centre for Genomic Sciences, Li Ka Shing Faculty of Medicine, The University of Hong Kong, Hong Kong SAR, China; 4 Department of Medical Genetics, The Capital Medical University, Beijing, China; 5 National Research Institute of Family Planning, Beijing, China

## Abstract

Inherited neuropathies show considerable heterogeneity in clinical manifestations and genetic etiologies, and are therefore often difficult to diagnose. Whole-exome sequencing (WES) has been widely adopted to make definite diagnosis of unclear conditions, with proven efficacy in optimizing patients’ management. In this study, a large Chinese kindred segregating autosomal dominant polyneuropathy with incomplete penetrance was ascertained through a patient who was initially diagnosed as Charcot-Marie-Tooth disease. To investigate the genetic cause, forty-six living family members were genotyped by SNP microarrays, and one confirmed patient was subject to WES. Through systematic computational prioritization, we identified a missense mutation c.G148T in *TTR* gene which results in a p.V50L substitution known to cause transthyretin-related familial amyloid polyneuropathy. Co-segregation analysis and clinical follow-up confirmed the new diagnosis, which suggested new therapeutic options to the patients and informed high risk family members. This study confirms WES as a powerful tool in translational medicine, and further demostrates the practical utility of gene prioritization in narrowing the scope of causative mutation.

Inherited neuropathies are a group of related disorders with debilitating conditions, including sensory, motor, and autonomic neuropathies. The most common type is Charcot-Marie-Tooth (CMT), or hereditary motor and sensory neuropathies^1^. The clinical classification is difficult due to overlapping symptoms, and it requires insensitive evaluations by experienced neurologists^2^, which may not always be practical in routine clinics^3^. Neuropathy can also be part of more generalized multisystem syndrome^1^, adding complexity to phenotype evaluation. It is therefore of interest to identify molecular genetic causes, which allows for prognosis, aids the family planning, or even suggests personalized treatment. However, genetic diagnosis of inherited neuropathy was traditionally hindered by its genetic heterogeneity, with at least 80 disease genes identified so far^1^,^4^.

Whole exome sequencing (WES) has now been widely adopted in molecular diagnosis of Mendelian disorders^5^,^6^, and has been shown to resolve undiagnosed conditions^7^ and to rescue misdiagnosis in some cases^8^. The ability to survey the entire protein coding region of the genome makes WES ideally suitable for disease like inherited neuropathy, which shows locus heterogeneity and wide phenotype spectrum^9^,^10^,^11^,^12^. Rapidly narrowing down the scope of disease-causing mutations is the key to make a timely genetic diagnosis^13^. Recent large-scale investigations typically used a combination of disease-specific knowledgebase^14^, inheritance mode^15^, and bioinformatics prediction of deleteriousness^16^. They also showed that the diagnostic yield for autosomal dominant cases was lower than average^16^,^17^,^18^, partly because of the difficulty in sifting through large number of private variants especially when only one single patient was sequenced. Computational methods for gene prioritization have undergone continuous development^19^; but their utility in analyzing clinical WES data remained to be demonstrated.

In the present study, we investigated the genetic cause of polyneuropathy within a large Chinese kindred ascertained from a patient initially diagnosed as CMT disease. After excluding the causes of known CMT genes, we performed WES in a single patient and applied computational prioritization to rank rare functional variants. We report the use of this approach to rescue the initial misdiagnosis in the proband and suggest new treatment options to the patients.

## Results

### Clinical Summary

The proband (III:21) was a 44-year-old man, presenting with spreading progressive limbs weakness and paresthesia. The patient initially felt mild weakness in lower limbs after labor work ten years before admission to clinic. Five years ago, he began to felt temperature loss and numbness in his right middle toe, which gradually progressed to knee joints and hands in a symmetrical fashion. Intermittent diarrhea without abdominal pain or vomiting occurred three years ago followed by severe weight loss, but no abnormality was found by colonoscopy examination. Around the same time, he started to develop severe weakness in limbs and was no longer competent for physical labor. Two years ago, he presented frequent syncope which occurred mostly at standing position. Urinary and fecal incontinence, *impotentia erigendi* were also noticed shortly.

**Figure 1:**
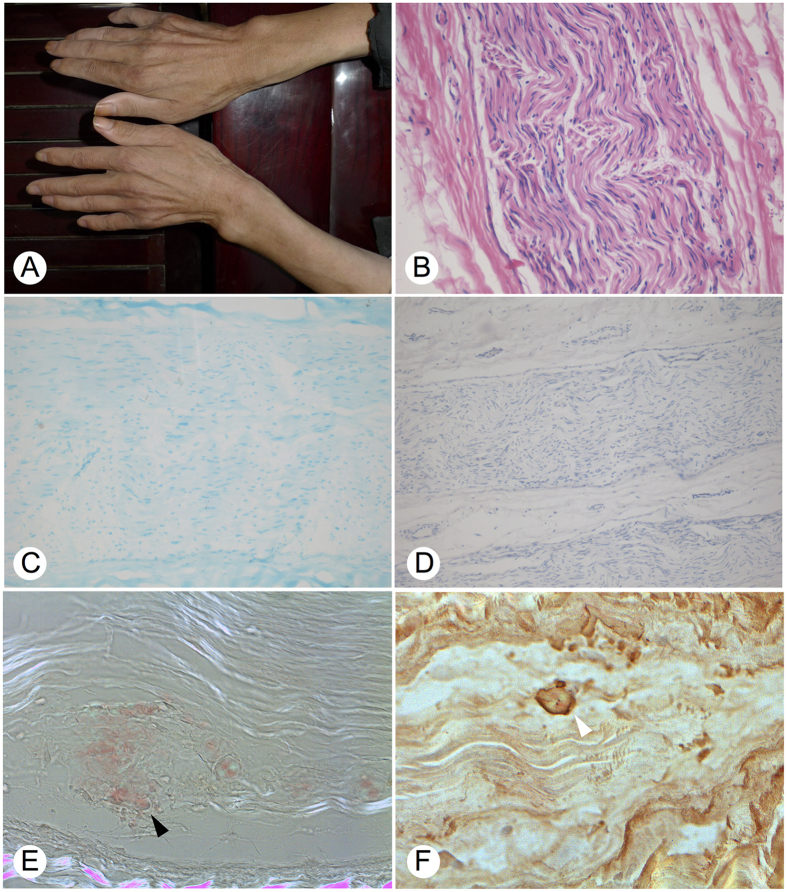
Clinical and pathological features of the proband. (**A**) Muscular atrophy observed in both hands. (**B**) Hematoxylin and eosin stain of sural nerve biopsy specimens suggests the absence of inflammatory cell infiltration, no regeneration of nerve plexus, and no amorphous deposits of amyloid substance. (**C**) Luxol fast blue stain reveals markedly decreased density of myelinated nerve fibers. (**D**) Congo red stain under bright light fails to detect amyloid deposit. (**E**) Congo red stain of the second sural nerve biopsy shows scattered endoneural amyloid deposit (black arrow) (**F**) Immunohistochemistry was carried out on paraffin sections with a polyclonal antibody to TTR. Amyloid deposits appeared as brown staining beside the nerve fiber bundles (white arrow).

In physical examination, right *pes cavus* and bilateral foot drop were observed. Blood pressure (BP) measurements revealed orthostatic hypotension (systolic/diastolic BP: 115/68 mmHg in recumbent and 50/30 mmHg in standing position). Neurological examination showed slight muscle weakness (5-/4, MRC scale) in the upper and lower extremities with muscular atrophy (Fig. 1A). Abdominal, cremasteric, plantar, and deep tendon reflexes were all absent. Sensory examination showed severe loss of pin-prick, temperature, and light-touch perception predominantly in the distal part of four extremities. Vibration and position senses were also impaired.

Nerve condition studies (Supplementary Table S1) demonstrated that the velocity and compound muscle action potential (CMAP) of bilateral tibial nerve and common peroneal nerve could not be elicited. Similarly, sensory conduction velocity and sensory nerve action potential (SNAP) of tibial, sural, and common peroneal nerves in the lower limbs could also not be detected. Both CMAP and SNAP of median and ulnar nerves showed decreased amplitudes and prolonged distal latency. Electromyogram exam showed neurogenic damage at the first dorsal interosseous muscle and left tibial anterior muscle. Echocardiography exam showed left ventricular hypertrophy, diffuse thickening of the left ventricular wall, and decreased diastolic function of the left ventricle. A biopsy of left sural nerve was carried out and showed an almost complete loss of myelinated fibers (Fig. 1C), but testing of amyloidosis was negative (Fig. 1B,D).

The proband reported that his father was suffered from similar disease symptoms on age of 40 s and died 10 years later due to malnutrition. An initial diagnosis of CMT disease was suggested by the referral neurologists.

### Genetic Findings

**Figure 2:**
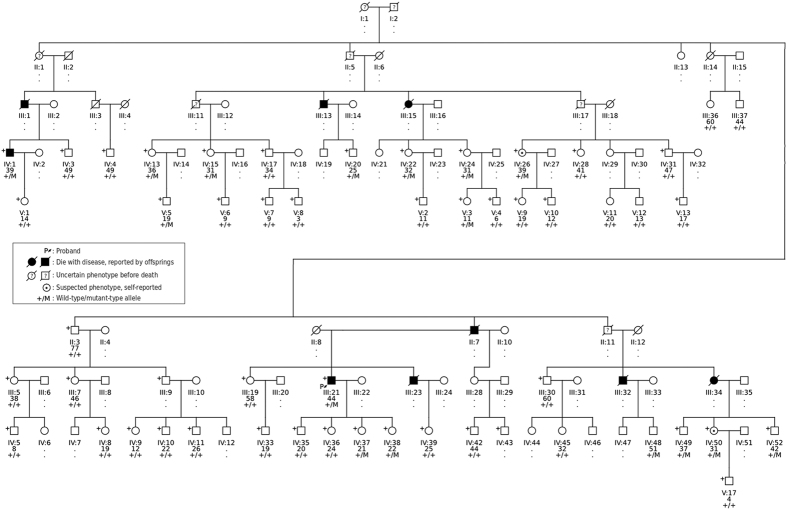
The pedigree structure of the full kindred. The neuropathy symptom was typically late on-set (~40 yrs.) in the family and may have incomplete penetrance. Most living family members were in the fourth or fifth generation of the pedigree. Two of them, IV:1 and III:21 (proband), received a definite diagnosis of sensory-motor neuropathy. Only one affected subject (IV:1) had sufficient DNA quantity for inclusion in exome sequencing. Forty-six individuals with SNP genotypes available are indicated by “+” and are included in pedigree analysis. Individuals with available DNA were later genotyped for the disease causing mutation *TTR*: p.V50L. The sanger sequencing traces of selected samples are shown in Supplementary Figure S4. Below each symbol shows individual’s identifier, age, and mutation genotypes.

The entire kindred was subsequently ascertained from a village in northern Henan Province, which spanned five generations (Fig. 2). A total 51 voluntary family members participated in the study and donated blood samples.

The disease was transmitted as an autosomal dominant trait in the pedigree, with reduced penetrance. Most living family members were in the fourth and fifth generation and were unaffected; but many of them reported that one of their parents died from the disease. Based on self-reported symptoms followed by clinical evaluation, we could only confirm one other affected subject (IV:1) with similar sensory-motor neuropathy.

**Figure 3:**
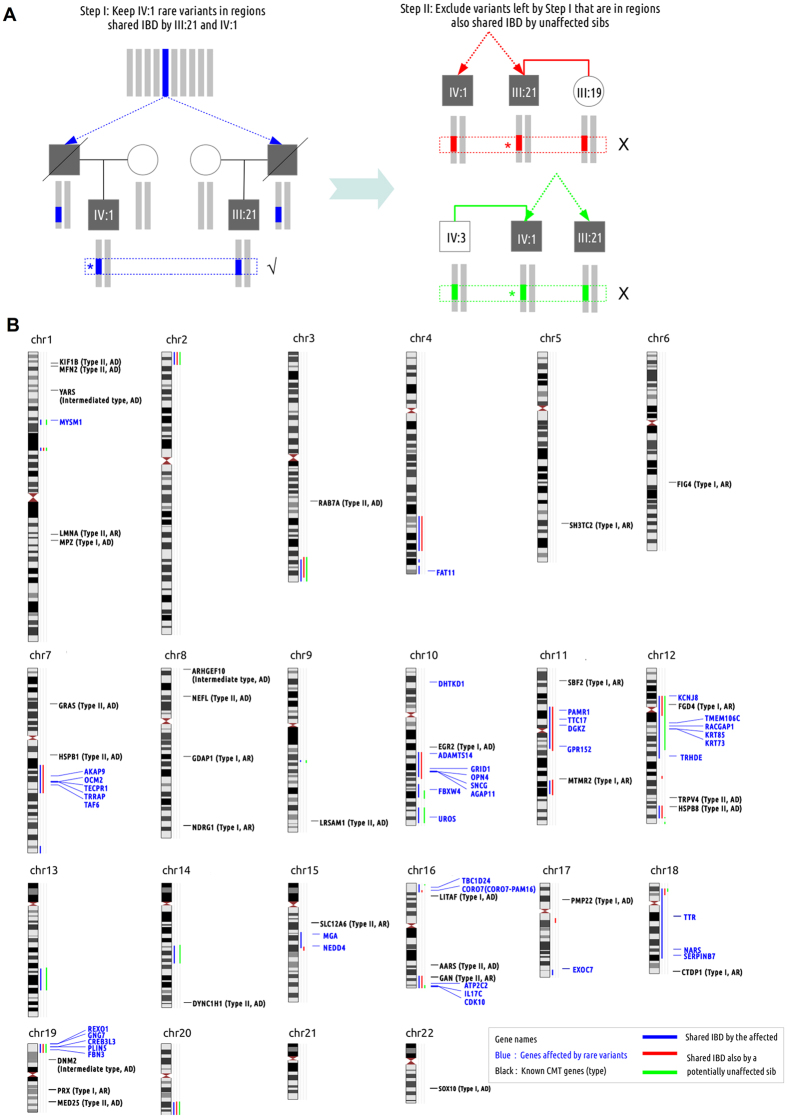
Identity-by-descent (IBD) filtering strategy and genome-wide IBD shared regions. (**A**) For the two confirmed patients, the disease causing variant should reside on the haplotypes shared IBD from their common ancestor. So we first keep only rare variants in genomic regions shared IBD by the patients. Both patients also have an older but unaffected sib. Assuming the sibs are not mutation carriers, they should not carry the haplotypes shared IBD with both patients. Therefore, we further exclude variants in regions shared IBD by both patients and at least one sib. (**B**) The whole-genome overview of IBD shared regions, locations of known Charcot-Marie-Tooth disease genes, and genes affected by ultra-rare functional variants discovered from one patient’s (IV:1) exome.

Forty-six samples were genotyped on ~5000 informative SNPs genome wide. Although it is not possible to map the disease locus by linkage analysis in this pedigree, the scope of disease causing allele should be confined to the extended haplotype shared identical-by-descent (IBD) by the two patients (Fig. 3A, left). We inferred 26 such shared IBD regions from genotypes, which scattered on 16 chromosomes (Fig. 3B, blue bars). By intersecting those regions with 35 known CMT genes (Supplementary Table S2), we could found only one autosomal dominant CMT gene (*HSPB8*), but Sanger sequencing on this gene did not reveal any non-synonymous variant.

After excluding the cause of known CMT genes, we performed WES. Although it would be ideal to sequence two distantly related patients (kinship coefficient = 1/32), only one subject (IV:1) had enough DNA for exome capture. A total of 14.5 Giga base pairs raw (70 bp paired-end) sequence reads were generated. Over 85% of reads can be aligned to the genome with high mapping quality (MAPQ>10). 88% of the targeted regions were covered by at least 20 non-redundant reads (and 93% of targets covered by 10×) with an average sequencing depth of 141×. After a series of filtering (Materials and Methods), we identified 236 ultra-rare functional variants affecting 228 genes, more than half of which were completely absent from controls (Supplementary Table S3). The number could be reduced to 42 variants of 42 genes, in regions shared IBD by the two patients (Fig. 3B).

We took two complementary strategies to narrow the scope of causal variant. First, we noted that both patients also had healthy sibs (III:19 and IV:3), who was older than the patient (Fig. 1). If we assume the sibs did not carry the causal variant, then the shared IBD regions can be excluded if they are also shared IBD by III:19 or IV:3 (Fig. 3A, right). The number of ultra-rare variants can then be reduced to 13 (Fig. 3B, Supplementary Table S3). One potential caveat to the above approach is that the causal variant has reduced penetrance also carried by the unaffected sibs.

As a complementary strategy, we adopted a bioinformatics prioritization approach to rank the 42 genes affected by the rare variants. We hypothesized that for inherited neuropathies, different disease genes were functionally related to each other, and potential disease gene in this pedigree could be distinguished from others by their functional relationship to known CMT genes (“seeds”). We implemented a method that ranked genes based on their functional relatedness to seeds in the gene network^20^ (Materials and Methods). The seeds were defined as 45 known and candidate CMT genes^21^ (Supplementary Table S2). In the cross-validation test, we found this method could prioritize the known CMT genes from a random background at the top 20% best scoring genes in more than 98% prioritization processes (Supplementary Figure S1).

We applied the prioritization method and intersected the 20% top ranked genes with 14 variants survived after IBD-based filter, four variants were highlighted and shown in Table 1. The missense single nucleotide variant (SNV) of top ranked gene *NARS* was first excluded, because it also appeared in Asians from 1000 Genomes Project and was likely a low-frequency polymorphism. The remaining private variants were first genotyped in proband (III:21); we then performed haplotype analysis to infer the carrier status in the kindred. The frameshift insertion in *NEDD4* gene was also excluded because it was not shared by the proband and likely inherited from an unaffected mother (Supplementary Figure 2B).

By manual review of the two remaining prioritized variants, we noted a missense SNV *TTR*(NM_00371.3):c.G148T in exon 2 of the gene encoding transthyretin, which results in a Valine-for-Lysine substitution at 50^th^ amino acid position. The same amino acid substitution was previously reported as a pathogenic variant causing transthyretin-related familial amyloid polyneuropathy in a Japanese family (TTR-FAP; OMIM:105210)^22^. The genetic result thus led to a new diagnosis different from initial suggestion.

### Validation and Follow-up

To validate the finding, we genotyped the two private missense SNVs in all participants. The segregation patterns in the pedigree were consistent with the haplotype analysis on overlapping samples (Supplementary Figure 2A,C). The *TTR* variant segregates into all branches in which offspring reported neuropathy in deceased parents (Fig. 1), whereas the segregation of the *MGA* variant cannot fully explain the parental phenotypes (Supplementary Figure S3).

The proband’s onset age at third to fourth decade, sensory-motor neuropathy with autonomic dysfunction at the full development, and his father’s death 10 years after symptom’s onset were all well matched to the characteristic phenotypes of TTR-FAP, except for the amyloid-negative biopsy. A second nerve sural biopsy was attempted after the identification of the *TTR* variant. Congo red staining confirmed the presence of amyloid in the endoneurium of multiple fascicules (Fig. 1E). TTR-immunolabeling showed strong immunoreactivity (Fig. 1F).

## Discussion

Transthyretin is a homo-tetrameric plasma transport protein for thyroid hormones and retinol, mostly produced in liver. Mutations in *TTR* gene cause the protein to misfold into amyloidogenic species, whose aggregation in extracellular in the peripheral nervous system and other organs causes life-threatening TTR-FAP. The accumulation of misassembled amyloid fibrils can cause damage to nerves by mechanical compression, blood vessel invasion, and direct toxic effects^23^.

To date, more than 100 disease causing mutations have been reported, the most frequent being the p.V50M caused by c.G148A substitution, which is a known SNP (rs28933979) located within a CpG dinucleotide sequence context. This mutation shows highest frequencies in Portugal, Sweden and Japan, possibly due to recurrent ancestral substitution in founder populations^24^,^25^. It also explains the relative high prevalence of FAP-TTR in those countries as compared with elsewhere in the world. The disease causing SNV identified in this study co-localizes with this mutational hotspot, but with a novel allele c.G148T (p.V50L), which also differs from previous report in the Japanese family c.G148C causing the same amino acid change^22^.

Patients with TTR-FAP may experience very different patterns of neuropathies which pose a challenge for diagnosis^26^. Common causes for delayed diagnosis or misdiagnosis include atypical presentation of nerve conduction changes, mild or subclinical dysautonomia, lack of family history, and negative amyloid test in biopsy^27^,^28^,^29^. Failure to recognize the disease symptoms due to low prevalence in China and misleading results from Congo red staining due to its medium sensitivity are the two major causes of initial misdiagnosis in our case.

Liver transplantation, which allows suppression of the mutant TTR production, can stop the disease progression and increase survival time^30^. Tafamidis, a kinetic TTR stabilizer, was also demonstrated to slow the progress of peripheral neuropathy, and has obtained marketing authorization in Europe^31^. Both therapies have been suggested to the patients. However, they are proven more effective for patients in early stages of the disease^30^,^32^, emphasizing the urgent need for early diagnosis aided by sequencing.

Both the age of onset and penetrance of TTR-FAP are known to vary to a high degree, and were thought to be the result of both genetic and non-genetic modifying factors^33^,^34^,^35^,^36^. The variability can even be observed within the same pedigree. In our kindred, both patients had disease onset in their 30 s. By direct sequencing, we also identified 14 other mutation carriers (Fig. 2), most of them have yet to reach the onset age. Among the mutation carriers, there were two suspected cases with self-reported feet discomfort (IV:25, IV:50) in their 30 s; but we also observed two apparent healthy adults (IV:48, IV:52) who were over 40 years old. Extensive medical monitoring was advised for pre-symptomatic mutation carriers, following the latest recommendation^37^.

It is interesting to note that we started this project aiming at finding new CMT gene, but WES ultimately led to the identification of causal variant in a different but related disease. WES has been successfully applied in a number of cases to correct initial misdiagnosis. In Supplementary Table S4, we provide an incomplete survey of the literature. The most common cause for misdiagnosis is the atypical manifestation mimicking other related disorders. In all cases, the disease genes and causal variants were found by use of the inheritance information. This strategy is efficient for autosomal recessive or X-linked diseases, because a typical exome contains only a handful of homozygotes, compound heterozygotes, or X chromosome rare functional variants. Similarly for sporadic cases caused by dominant *de novo* functional mutations, sequencing trios can be very efficient to pinpoint the genetic etiology due to the low exome-wide mutation rate^38^. Autosomal dominant diseases, however, represent a difficult situation for gene discovery especially in small pedigrees. In our study, only one patient was eligible for sequencing due to technical reasons; but the situation is not uncommon in clinical laboratory. Both IBD-based filtering and gene prioritization were used to sift through rare functional variants. It is worth noting that even by using gene prioritization alone, the *TTR* gene is still ranked within top 10 list. Since a typical exome contains 100~200 rare functional variants^39^, it was a considerable reduction of the searching space.

Given the successful application of gene prioritization in our case, we also tested its efficacy in narrowing down the disease gene on reported misdiagnosed cases without relying on inheritance. For each reported case, the confirmed disease gene was combined with 199 randomly selected ones across the genome (with probability of being selected proportion to the coding sequence length to mimic the genes affected by rare functional variants). Then we used the known and candidate genes of the disease suggested by the initial diagnosis to form seed genes. The candidate genes were ranked by their functional relatedness to the seed genes. The overall process was repeated 1000 times. In all except one cases, the average rank of the confirmed disease gene was within top 10% (Table 2), which is feasible for manual review in practice. One exceptional case is the discovery of novel phenotype to a known syndrome that was not observed before. The results is an empirical demonstration that underlying genes responsible for phenotypically similar diseases are functionally related^40^. We therefore advocate the application of gene prioritization in clinical WES to search for genetic causes beyond initial suspect, especially when the inheritance mode is unclear.

Together, our findings in this work confirm power of WES as a clinical diagnostic tool and showcase the effectiveness of computational gene prioritization.

## Materials and Methods

### Ethics Statement

This research project was approved by the ethical committee of National Research Institute of Family Planning. The methods and experiments were carried out in accordance with approved guidelines. Written informed consent was obtained from all participants.

### Clinical Assessment

The proband had undergone a standard neurological examination including muscle strength, tendon reflex testing, and evaluation of light touch, pinprick, vibratory and temperature sensations, and position sense. Autonomic involvement was assessed by recording the presence/absence of nausea, vomiting, diarrhea, constipation, urinary and stool incontinence, impotence etc. Measurements of blood pressures and pulse rate were taken in the recumbent and standing positions. Needle electromyography recording were performed using standard concentric needle electrode. Motor and sensory nerve conduction studies were performed by surface stimulation and recording electrodes using standard techniques. Additional laboratory tests were performed to exclude infectious, malignancies, or autoimmune disorders. A sural nerve biopsy was carried out after obtaining informed consent. The formaldehyde-fixed paraffin-embedded pathological specimen was examined with hematoxylin-eosin, Luxol fast blue, Congo red stains using standard protocols. Immunolabeling was carried out with polyclonal rabbit antibodies to transthyretin,

### Exome Sequencing and Data Processing

Exome sequences of the selected subject (IV:1) were enriched from genomic DNAs using Agilent SureSelect V2 whole exome kit. After hybridization, captured DNA was amplified and sequenced on Illumina Genome Analyzer IIx with 70 bp paired-end reads following manufacturer’s protocol.

We used BWA v0.59 (3) to align sequence reads to the human genome reference (build 37) and removed duplicated reads from subsequent analyses. We used GATK v1.4–9^41^ to realign the mapped reads around known and potential indels and recalibrated base quality score before calling variants. The variant calls were then subject to quality controls following GATK’s recommended parameters. We filtered QC-passed variants to exclude those having allele frequencies >=0.5% in dbSNP135, 1000 Genomes Project (March 2012 release), and our in-house databases (180 unrelated exomes of Chinese ancestry processed by the same computational pipeline). We then annotated remaining variants to genes using Variant Effect Predictor^42^ and kept only missense, nonsense, frameshift, non-frameshift, or splice site variants. Single nucleotide variants were further filtered to remove sites that were not evolutionary conserved, with genome evolutionary profiling score GERP<2.0^43^. The remaining ultra-rare functional variants were used in subsequent analysis.

### Pedigree Analysis

Forty six family members were genotyped by Illumina Linkage-24 SNP array which contains 5,566 autosomal SNP markers with high information content. Genotypes were called by Illumina BeadStudio (v3.0). The raw genotypes were filtered to exclude SNPs that were monomorphic or had more than 10% missing genotypes in the pedigree. Pairwise sample relationships were verified using RELPAIR^44^; and likely genotyping errors were identified and masked by PedCheck^45^ and Merlin^46^. The resulting 4,876 high quality SNPs were used for subsequent analyses. Genetic positions were linear interpolated from the fine-scale recombination map^47^.

To accommodate large pedigree size, we partitioned the entire pedigree into overlapping subsets. IBD analysis was performed using merlin^46^ which calculated probabilities of sharing 0, 1, or 2 alleles IBD for each pair of individuals at each marker position. The shared IBD segments were then defined by the regions spanned by the SNP markers with Prob(IBD = 1) + Prob(IBD = 2) > 0.5. To perform haplotype analysis, we extracted the SNP genotypes ~10 cM around the candidate variants. We used merlin to infer most probable haplotypes in each sub-pedigrees, and then merged those subsets based on the consensus haplotypes on overlapping samples. Pedigree was drawn by Madeline 2.0^48^ and edited by Inkscape software. Haplotypes were graphically visualized using HaploPainter^49^.

### Gene Prioritization

The underlying assumption for prioritization is that, for a genetically heterogeneous disorder (or a group of related disorders), disease genes are functionally related to each other. If we have already identified a representative subset of the disease genes (or “seed genes”), potential disease genes could be distinguished from other unrelated ones based on their functional relatedness to the seed genes.

To quantify the functional relationship, we used protein network database STRING v9.0^50^. The functional association network was first converted to a weighted undirected graph represented by an adjacency matrix **W**. We then normalized **W** to obtain a transition probabilities matrix **A** = **D**^−1/2^**WD**^−1/2^, where **D** is a diagonal matrix with 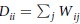. Following Kohler, *et al*.^20^, the functional relationship between a gene to seed genes can be defined as the arrival probability in steady states of random walk with restart from seed genes. The process can be described by


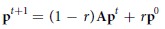


where **p**^*t*^ is a is a vector with *i*-th element representing the probability of arriving at gene *i* at step *t*. The probability vector at time 0 (**p**^0^) is initialized to hold 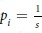 for seed genes (where *s* the number of seed genes), and 0 otherwise. Parameter *r* represents the probability of restarting from the seed genes and was set to 0.5. The steady state was achieved when 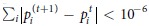. The steady state probability then reflect a gene’s functional relatedness to seed genes.

The prioritization performance was assessed by a leave-one-out cross validation (LOOCV) test. In LOOCV, each known disease gene is, in turn removed from the seeds and added to a set of 199 randomly selected genes to create an artificial candidate set. Then we used the remaining known disease genes as seeds; and calculated functional relatedness of candidate genes to the seeds. The candidates were then ranked by their scores. The rank of the left-out seed gene among other 199 random genes thus reflected the discriminative ability of the score to distinguish true disease genes from random background. After each seed gene and their associated random sets were scored in this way, we calculated the chances that the left-out known disease gene can be found at different rank cutoffs (top 5%,, 10%, 20%, …). The detection rate (sensitivity) were plotted against different cutoffs (1-specificity), resulting in a Receiver Operating Characteristic (ROC) curve.

## Additional Information

**How to cite this article**: Chen, H. *et al*. Exome Sequencing and Gene Prioritization Correct Misdiagnosis in a Chinese Kindred with Familial Amyloid Polyneuropathy. *Sci. Rep*. **6**, 26362; doi: 10.1038/srep26362 (2016).

## Supplementary Material

Supplementary Information

## Figures and Tables

**Table 1:** Ultra-rare functional variants found in one affected subject (IV:1) prioritized by two complementary strategies.

Gene*	Variants	Shared by Proband	Functional effects	cDNA change	Protein change	Grantham score	Non-synonymous SNV Predictions†	PhyloP‡	GERP^‡^
*NARS*§	18:55269699 T>C	N.A.	Missense SNV	NM_004539.3:exon13: c.A1403G	p.N468S	46	+ ? + +	5.14	6.16
*TTR*	18:29172937 G>T	Y	Missense SNV	NM_000371.3:exon2: c.G148T	p.V50L	32	− − + +	3.02	5.71
*NEDD4*	15:56207937 C>CA	N	Frame-shift insertion	NM_198400.2:exon1: c.1093_1094insT	p.V365Cfs*3	N.A.			
*MGA*	15: 42005407 A>G	Y	Missense SNV	NM_001164273.1:exon24: c.3143A>G;	p.N1048S	46	+ + ? ?	1.87	5.75

^*^Genes are ranked based on their functional relatedness to known and candidate CMT genes.

^†^Results from four non-synonymous SNV effect prediction algorithms, from left to right: PolyPhen2, SIFT, LRT, MutationTaster. +: deleterious or damaging, -: benign; ?: unknown.

^‡^Measures of evolutionary constraint. PhyloP is the −log10 of p-value for testing the null hypothesis of neutral evolution, based on 46-way whole-genome alignment of vertebrates. Genome evolutionary rate profiling (GERP) score can be interpreted as the substitutions expected under neutrality minus the number of substitutions observed at the position, which was derived from 35-way whole-genome alignment of mammals and had a theoretical maximum of 6.18.

^§^The missense variants in *NARS* gene also appeared once in Asian population of 1000 Genomes Project.

**Table 2:** Application of gene prioritization to previously reported cases.

Reference	Disease gene identified by sequencing	Genetic diagnosis	Candidate genes for the disease of initial diagnosis	Average rank of disease gene	Percentage in top 10% best scoring genes
Choi *et al*.^51^	*SLC26A3*	Congenital chloride diarrhea	*SLC12A1, SLC12A3, KCNJ1, CLCNKB, BSND, CLCNKA, CASR*	4.50%	100%
Majewski *et al*.^52^	*PEX1*	Peroxisome biogenesis disorder	*GUCY2D, RPE65, SPATA7, AIPL1, LCA5, RPGRIP1, CRX, CRB1, NMNAT1, CEP290, IMPDH1, RD3, RDH12, LRAT, TULP1, KCNJ13*	59.9%	0%
Worthey *et al*.^53^	*XIAP*	X-linked lymphoproliferative syndrome	*IKBKG, NOD2, HPLH1, UNC13D, PRF1, STX11, STXBP2, FOXP3, TNFSF6, TNFRSF6, CASP10, CASP8, NRAS, SH2D1A*	0.60%	100%
Chaudhry *et al*.^54^	*BSCL2*	Silver spastic paraplegia syndrome	*KIF1B, MFN2, LMNA, YARS, TRPV4, HSPB8, DYNC1H1, SLC12A6, AARS, GAN, DNM2, MED25, RAB7A, GARS, HSPB1, ARHGEF10, NEFL, LRSAM1*	5.0%	100%
Hanchard *et al*.^55^	*CACNA1S*	Atypical hypokalemic periodic paralysis	*SCN4A, PRRT2, KCNMA1, SLC2A1*	1.1%	100%
Lieber *et al*.^56^	*WFS1*	Wolfram syndrome	87 mitochondrial diseases genes^57^	9.8%	53%
Zhan *et al*.^58^	*ABCD1*	X-linked adrenoleukodystrophy	*ALT1, SPAST, NIPA1, KIAA0196, KIF5A, RTN2, HSPD1, REEP1, ZFYVE27, SPG11, SPG7, ZFYVE26, ERLIN2, SPG20, SPG21, B4GALNT1, DDHD1, KIF1A, FA2H, PNPLA6, GJC2, GBA2, KIAA0415, TECPR2, DDHD2, C12ORF65, CYP2U1*	6.10%	99.50%
The present study	*TTR*	Familial amyloid polyneuropathy	45 known and candidate CMT genes (Supplementary Table S2)	4.40%	100%
