# Supplementary material for: Exome Sequencing and Gene Prioritization Correct Misdiagnosis in a Chinese Kindred with Familial Amyloid Polyneuropathy
Source: Sci Rep. 2016 May 23;6:26362. doi: 10.1038/srep26362 (PMC4876459; doi:10.1038/srep26362)
Supplement: Supplementary Information [file srep26362-s1.doc]

# Exome Sequencing and Gene Prioritization Correct Misdiagnosis in a Chinese Kindred with Familial Amyloid Polyneuropathy

Hui Chen^1^*, Xueya Zhou^23^*, Jing Wang^4^*, Xi Wang^5^, Liyang Liu^2^, Shinan Wu^5^, Tengyan Li^5^, Si Chen^5^, Jingwen Yang^5^, Pak Chung Sham^3^, Guangming Zhu^1#^, Xuegong Zhang^2#^, Binbin Wang^5#^

## Supplementary Figures

Figure S1 The Receiver Operating Characteristic (ROC) curve of leave-one-out cross-validation test on prioritizing known and candidate Charcot-Marie-Tooth disease genes (Table S2). AUC: area under the ROC curve.


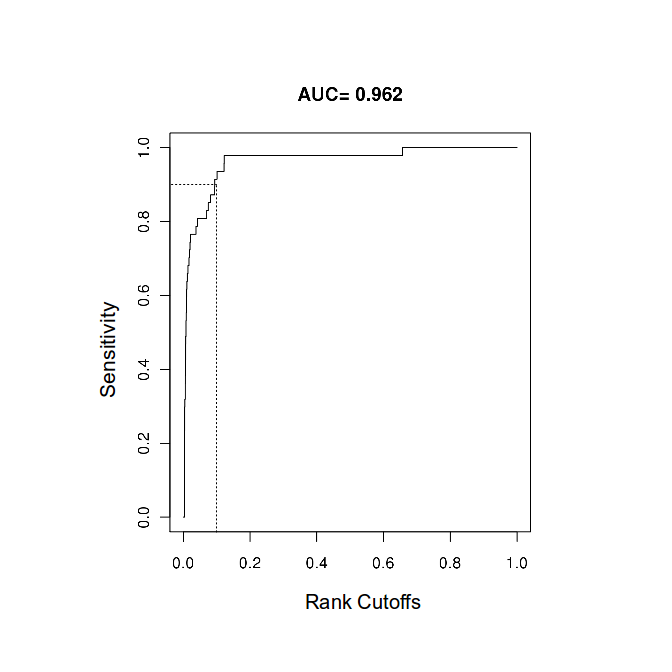


Figure S2 Haplotype sharing analysis on samples with SNP genotypes. For each candidate variant in Table 1 of main text, we reconstructed SNP haplotypes around 10 cM flanking regions on both sides. About 10 markers were selected for display. The position of variants relative to the marker positions are indicated by arrows. The haplotypes shared identity-by-descent by the two confirmed patients (III:21 and IV:1) are colored red; the remaining inferred founder haplotypes were colored in dark and light greys. In (A) *TTR* and (C) *MGA* cases, the variants identified in IV:1 were confirmed by Sanger sequencing in III:21, so red haplotypes are proxies of the variants. In (B) the *NEED4* case, Sanger sequencing revealed absence of the same variant in III:21, so the variant found in III:21 was inferred to be inherited from the maternal side (the green haplotypes), and not segregate into other branches of the pedigree.


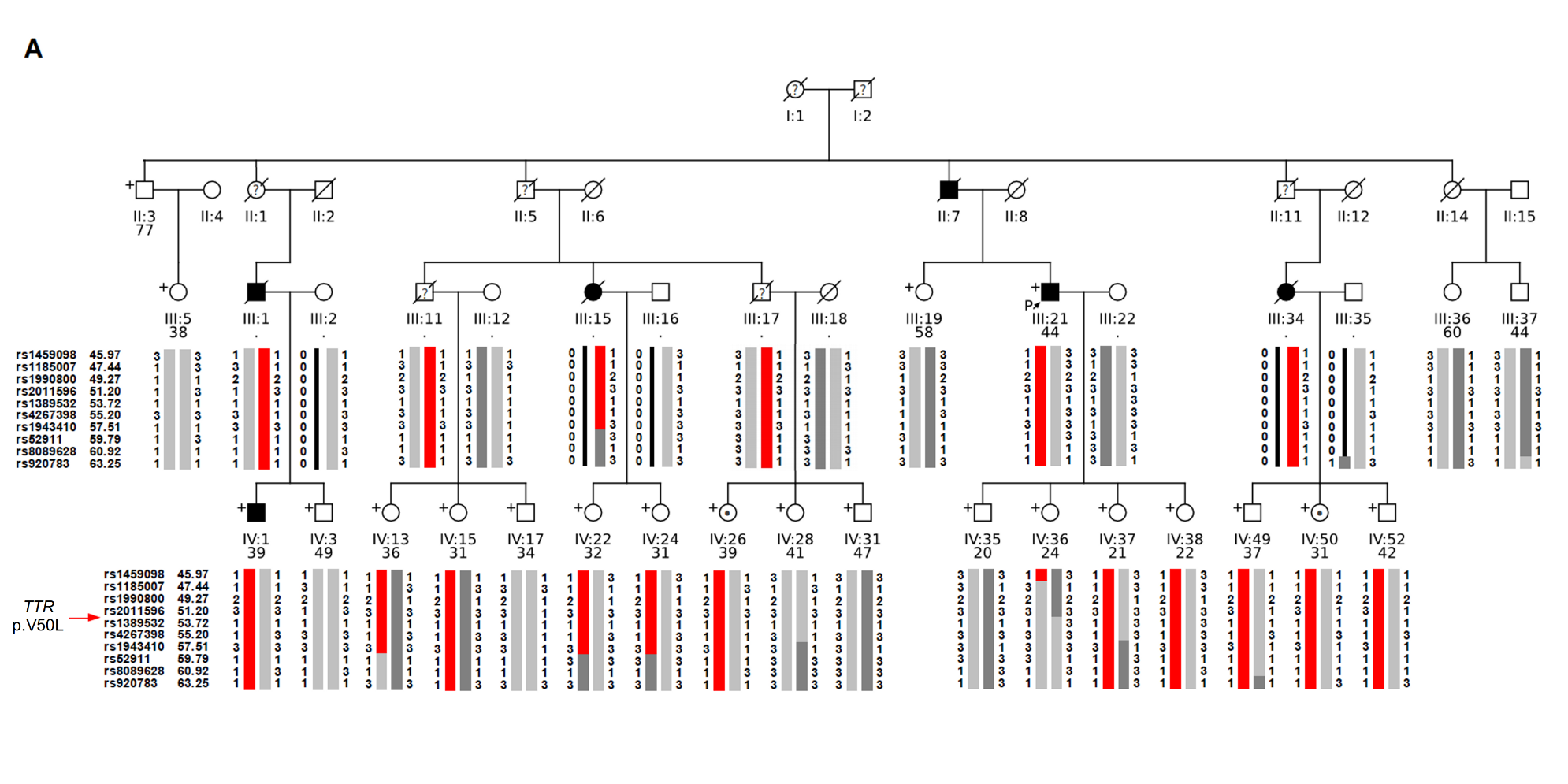


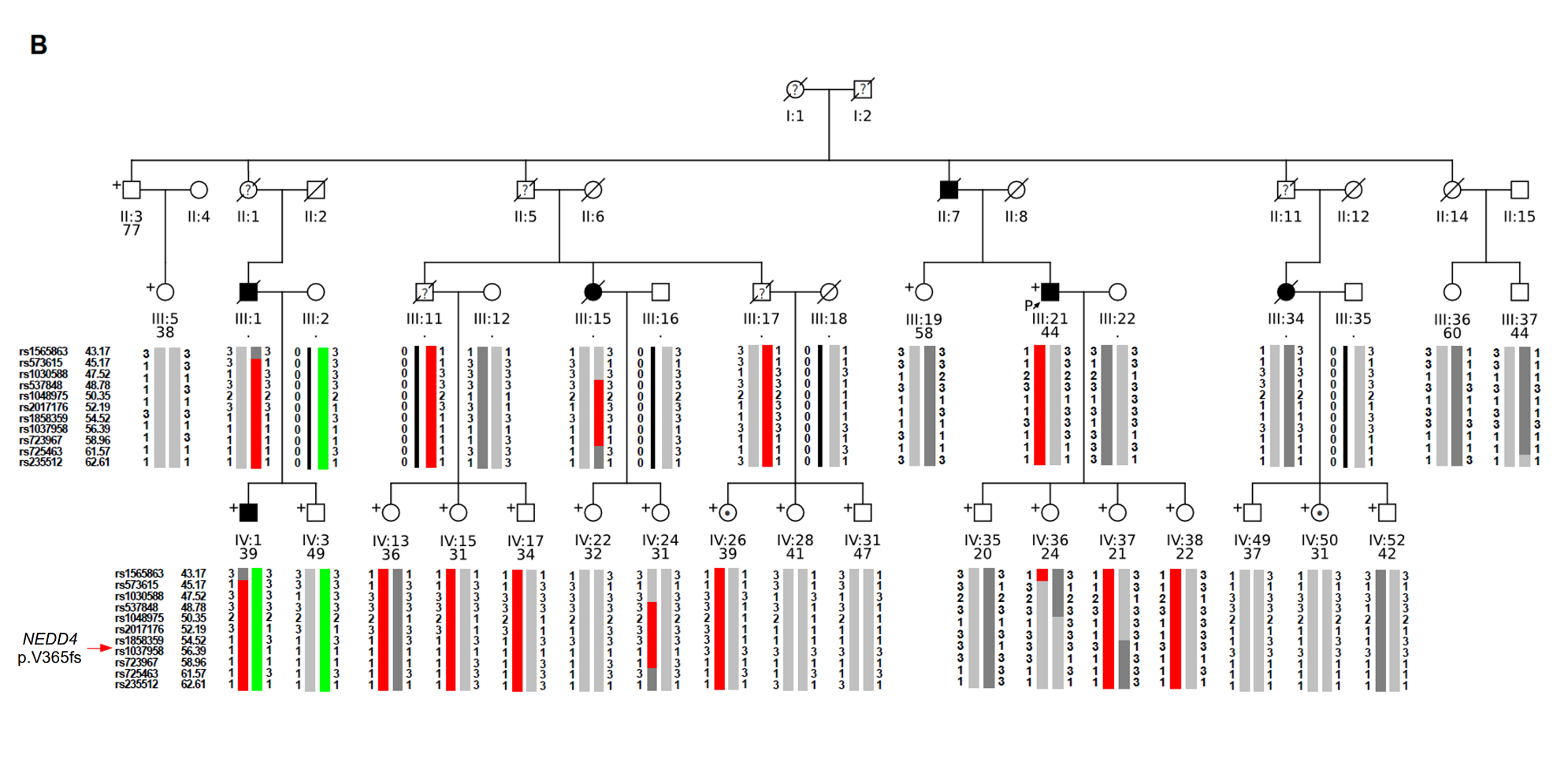

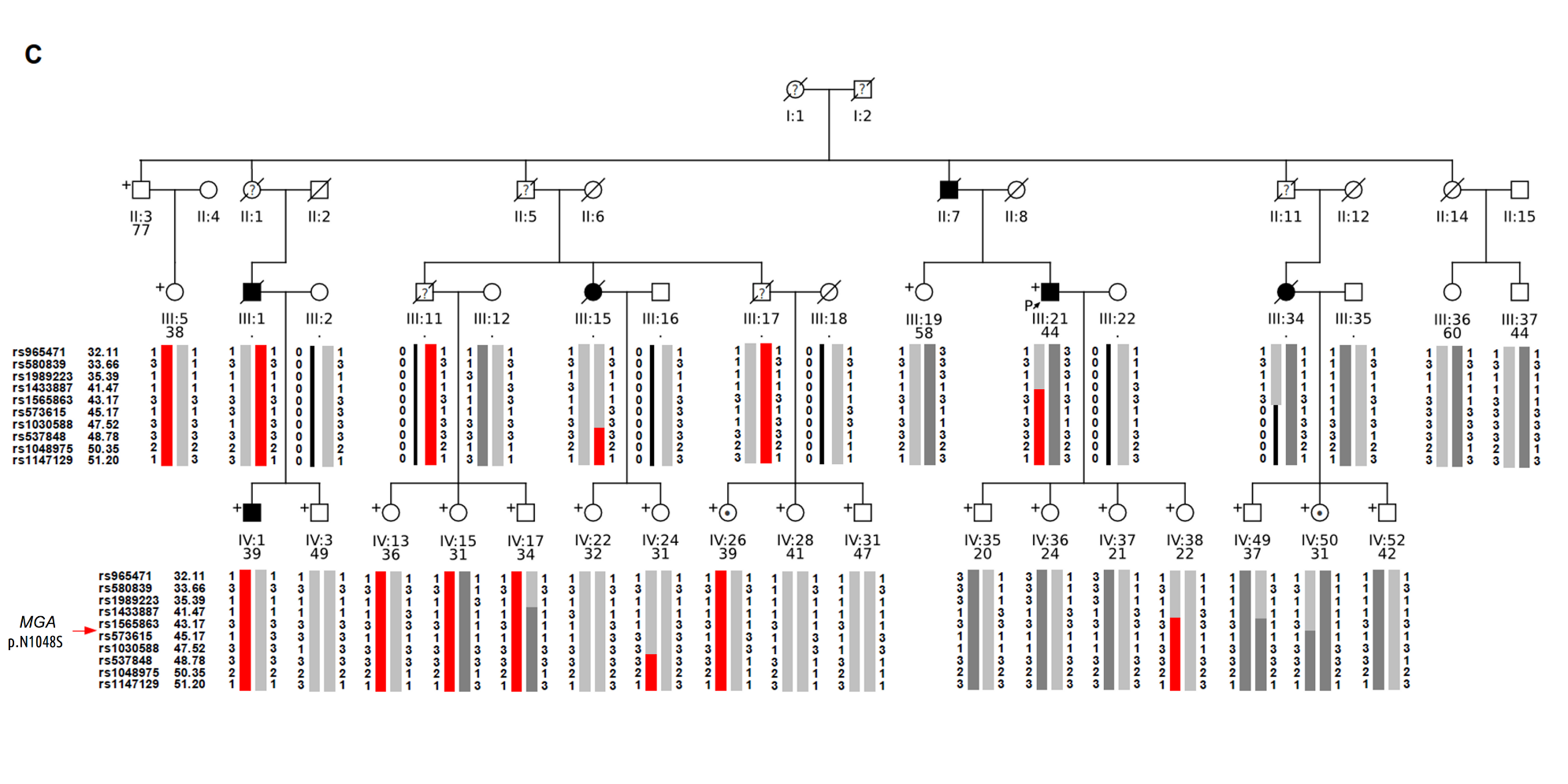


Figure S3 The co-segregation of *MGA* p.N1048S variant in the kindred. The pedigree symbols are the same as in Figure 2.


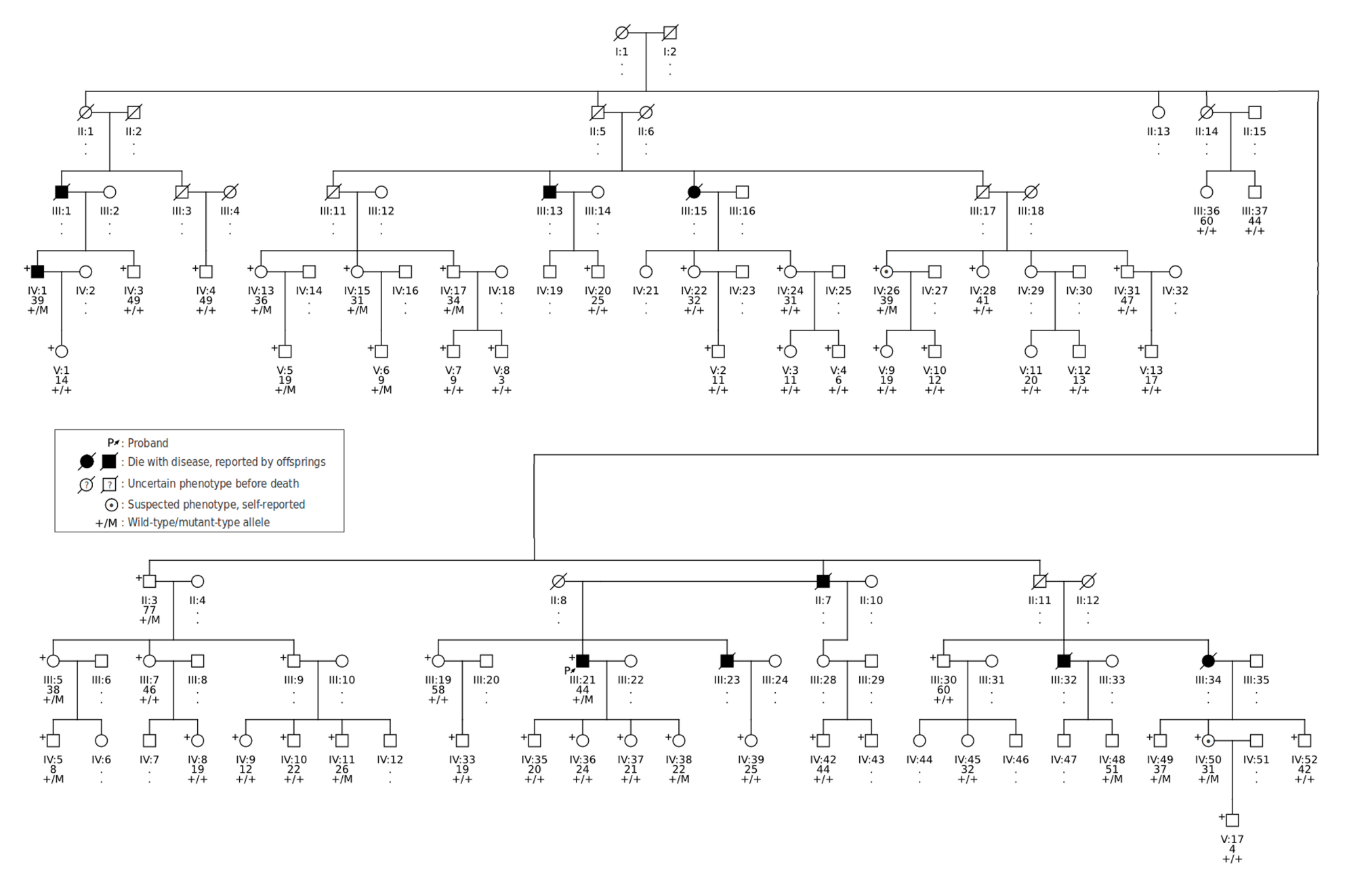


Figure S4 Sanger sequencing traces of the disease causing mutation *TTR*(NM_000371.3):c.G148T on selected individuals.


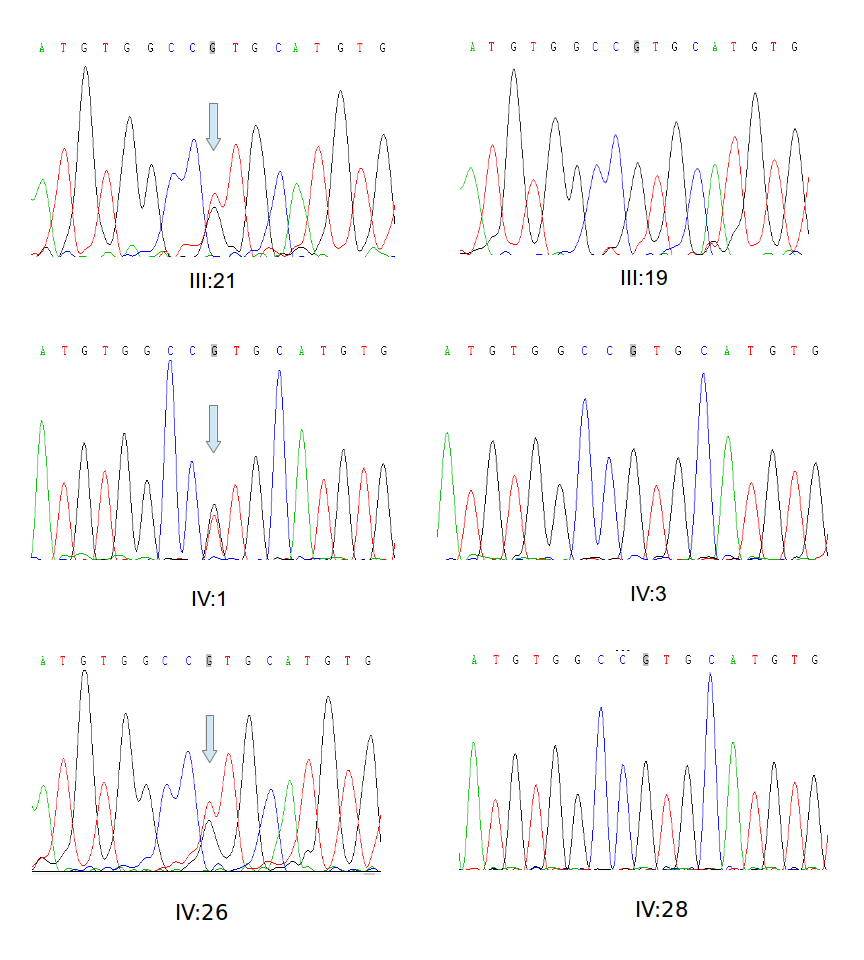


## Supplementary Tables

Table S1 Nerve conduction studies of the proband at the age of 44.

| Motor Nerve | Tract | MNCV(m/s) | | CMAP(mV) | | Latency (ms) | |
| --- | --- | --- | --- | --- | --- | --- | --- |
|  |  | Left | Right | Left | Right | Left | Right |
| Medianus N. | Wrist-APB | Absent | Absent | 1.02 | 0.90 | 2.85 | 2.90 |
|  | Elbow-Wrist | 38.3 | 33.5 | 1.04 | 0.68 | 8.59 | 9.46 |
|  | Erb’s pt-Elbow | 51.5 | 48.2 | 0.99 | 0.48 | 15.0 | 16.1 |
| Peroneus N. | Ankle-EDB | Absent | Absent | Absent | Absent | Absent | Absent |
|  | Fibular head-Ankle | Absent | Absent | Absent | Absent | Absent | Absent |
| Tibialis N. | Ankle-AHB | Absent | Absent | Absent | Absent | Absent | Absent |
|  | Knee-Ankle | Absent | Absent | Absent | Absent | Absent | Absent |
| Ulnaris N. | Wrist-ADM | Absent | Absent | Absent | 0.70 | 2.28 | 1.99 |
|  | Ab.elbow-Wrist | 37.5 | 37.8 | 0.80 | 0.45 | 8.94 | 8.61 |
|  | Erb’s pt-Ab.elbow | 62.0 | 63.4 | 0.47 | 0.48 | 14.1 | 13.5 |
| Sensory Nerve | Tract  (Normal Reference) | SNCV(m/s) | | SNAP(μV) | | Latency (ms) | |
|  |  | Left | Right | Left | Right | Left | Right |
| Medianus N. | Digit I-Wrist | 45.8 | 45.8 | 0.84 | 0.52 | 2.86 | 2.89 |
| Surallis N. | Ankle-Sural | Absent | Absent | Absent | Absent | Absent | Absent |
| Ulnaris N. | Digit IV-Wrist | 36.5 | 41.1 | 0.91 | 0.61 | 3.31 | 3.27 |

APB: abductor pollicis brevis; EDB: extensor digitorum brevis; AHB: abductor hallucis brevis; ADM: abductor digiti minimi; MNCV: motor nerve conduction velocity; CMAP: compound muscle action potential; SNCV: sensory nerve conduction velocity; SNAP: sensory nerve action potential.

Table S2 List of known and candidate CMT genes.

| Gene Symbol | Type and Inheritance |
| --- | --- |
| KIF1B | CMT2-AD |
| NGF | CAND |
| MFN2 | CMT2-AD |
| LMNA | CMT2-AR |
| NTRK1 | CAND |
| MPZ | CMT1-AD |
| YARS | CMT12-AD |
| PLEKHG5 | CAND |
| EGR2 | CMT1-AD |
| BSCL2 | CAND |
| IGHMBP2 | CAND |
| MTMR2 | CMT1-AR |
| SBF2 | CMT1-AR |
| TRPV4 | CMT2-AD |
| HSPB8 | CMT2-AD |
| FGD4 | CMT1-AR |
| HSN2 | CAND |
| DYNC1H1 | CMT2-AD |
| SLC12A6 | CMT2-AR |
| LITAF | CMT1-AD |
| AARS | CMT2-AD |
| GAN | CMT2-AR |
| PMP22 | CMT1-AD |
| SEPT9 | CAND |
| CTDP1 | CMT1-AR |
| DNM2 | CMT12-AD |
| PRX | CMT1-AR |
| MED25 | CMT2-AD |
| DCTN1 | CAND |
| SOX10 | CMT1-AD |
| RAB7A | CMT2-AD |
| SH3TC2 | CMT1-AR |
| FIG4 | CMT1-AR |
| GARS | CMT2-AD |
| HSPB1 | CMT2-AD |
| NDRG1 | CMT1-AR |
| ARHGEF10 | CMT12-AD |
| NEFL | CMT2-AD |
| GDAP1 | CMT1-AR |
| IKBKAP | CAND |
| LRSAM1 | CMT2-AD |
| SETX | CAND |
| SPTLC1 | CAND |
| PRPS1 | CMT1-X |
| GJB1 | CMT1-X |

CMT1/2: Charcot-Marie-Tooth neuropathy Type 1 and 2; CAND: candidate genes based on functional evidence or animal model; AD: autosomal dominant, AR: autosomal recessive; X: X-linked.

Table S3 The number of ultra-rare (or private) variants and their affected genes remained after each step of filtering.

| Filtering steps | Ultra-rare variants^¶^ | | | Private variantsǂ | | |
| --- | --- | --- | --- | --- | --- | --- |
|  | SNVs | Indels | Genes | SNVs | Indels | Genes |
| Keep conserved variants that change amino acid of encoded proteins or affect splicing | 222 | 14 | 228 | 127 | 10 | 135 |
| Keep variants that are located in regions shared IBD by two confirmed patients. | 37 | 5 | 42 | 22 | 5 | 27 |
| Exclude variants that are also shared by the unaffected siblings of patients | 12 | 1 | 14 | 7 | 1 | 9 |

¶ ultra-rare variants: allele frequency <0.5 in dbSNP 135, 1000 Genomes Project, and in-house database of 180 Chinese individuals; ǂ private variants: absent in dbSNP 135, 1000 Genomes Project and only appear once in in-house database.

Table S4 A survey of misdiagnosed cases rescued by exome sequencing.

| **Reference** | **Initial suspect** | **Mode of inheritance** | **Sequencing Strategy** | **Analytical strategy** | **Disease causing mutation** | **Genetic diagnosis** | **Reason for misdiagnosis** |
| --- | --- | --- | --- | --- | --- | --- | --- |
| [Choi, et al^. 1^](#_ENREF_1) | Bartter syndrome | AR | One patient | Homozygous variants in regions homozygous by descent identified by common SNPs | The homozygote of *SLC26A3*:p.D652N | congenital chloride diarrhea | Volume depletion was thought to be caused by renal salt-wasting |
| [Majewski, et al^. 2^](#_ENREF_2) | Leber congenital amaurosis | AR | One patient |  | The homozygote of *PEX1*:p.Gly843Asp | peroxisome biogenesis disorder in the Zellweger spectrum | Atypical (mild) representation of the syndrome |
| [Worthey, et al^. 3^](#_ENREF_3) | Several congenital immune deficiency syndromes | AR or X-linked | One patient | Genes harboring homozygous, hemizygous, or compound heterozygous mutations | The hemizygote of *XIAP*: p.C203Y | X-linked lymphoproliferative syndrome type 2 | Novel manifestation of the syndrome (Crohn disease-like illness) |
| [Chaudhry, et al^. 4^](#_ENREF_4) | Charcot-Marie-Tooth (CMT) hereditary neuropathy type 2, X-linked | X-linked | One patient | Variants in known peripheral neuropathy genes | The heterozygote of *BSCL2*:N88S | Silver spastic paraplegia syndrome,  distal hereditary motor neuronopathy type 5A, and CMT type 2 | Small pedigree and incomplete penetrance in patient’s mother |
| [Hanchard, et al^. 5^](#_ENREF_5) | Paroxysmal dyskinesia/dystonia; Atypical hypokalemic periodic paralysis (HypoKPP) | AD | The patient and her parents | De novo mutations  hypokalemic periodic paralysis | The de novo heterozygote of *CACNA1S*:R897S | Atypical HypoKPP resulting from the mutation of primary HypoKPP | Phenotype not fully consistent with primary hypokalemic periodic paralysis |
| [Lieber, et al^. 6^](#_ENREF_6) | Mitochondrial (MT) disorder | AR | One patient | Homozygous, hemizygous mutations in the genes implicated MT disorders or in diseases with multi-system phenotype | The homozygote of *WFS1*:p.R558C | Wolfram syndrome | Late onset of the typical phenotypes of Wolfram syndrome |
| [Zhan, et al^. 7^](#_ENREF_7) | Hereditary spastic paraplegia | AR or X-linked | Two affected siblings | Homozygous, hemizygous mutations, or compound heterozygous mutations shared by the siblings | The hemizygote of *ABCD1*:p.R554H | X-linked adrenoleukodystrophy | Pure spasticity without leukodystophic changes in brain MRI did not motivate the biochemical test. |

## References

1 Choi, M. *et al.* Genetic diagnosis by whole exome capture and massively parallel DNA sequencing. *Proceedings of the National Academy of Sciences of the United States of America* **106**, 19096-19101, doi:10.1073/pnas.0910672106 (2009).

2 Majewski, J. *et al.* A new ocular phenotype associated with an unexpected but known systemic disorder and mutation: novel use of genomic diagnostics and exome sequencing. *Journal of medical genetics* **48**, 593-596, doi:10.1136/jmedgenet-2011-100288 (2011).

3 Worthey, E. A. *et al.* Making a definitive diagnosis: successful clinical application of whole exome sequencing in a child with intractable inflammatory bowel disease. *Genetics in medicine : official journal of the American College of Medical Genetics* **13**, 255-262, doi:10.1097/GIM.0b013e3182088158 (2011).

4 Chaudhry, R. *et al.* Re-analysis of an original CMTX3 family using exome sequencing identifies a known BSCL2 mutation. *Muscle & nerve* **47**, 922-924, doi:10.1002/mus.23743 (2013).

5 Hanchard, N. A. *et al.* Exploring the utility of whole-exome sequencing as a diagnostic tool in a child with atypical episodic muscle weakness. *Clinical genetics* **83**, 457-461, doi:10.1111/j.1399-0004.2012.01951.x (2013).

6 Lieber, D. S. *et al.* Atypical case of Wolfram syndrome revealed through targeted exome sequencing in a patient with suspected mitochondrial disease. *BMC medical genetics* **13**, 3, doi:10.1186/1471-2350-13-3 (2012).

7 Zhan, Z. X. *et al.* Exome sequencing released a case of X-linked adrenoleukodystrophy mimicking recessive hereditary spastic paraplegia. *European journal of medical genetics* **56**, 375-378, doi:10.1016/j.ejmg.2013.04.008 (2013).
